# Widespread Multidrug Resistance and Virulence Determinants in *Escherichia coli* Across the Interconnected Farm-to-Food Continuum

**DOI:** 10.3390/antibiotics15050455

**Published:** 2026-04-30

**Authors:** David Yembilla Yamik, Wattana Pelyuntha, Wichanan Wannasrichan, Nattha Vigad, Kridda Chukiatsiri, Viphavanh Chanthavong, Mingkwan Yingkajorn, Kitiya Vongkamjan

**Affiliations:** 1 Department of Biotechnology, Faculty of Agro-Industry, Kasetsart University, Bangkok 10900, Thailand; davidyembilla.y@ku.th (D.Y.Y.); wichanan.wan1991@gmail.com (W.W.); 2 Futuristic Science Research Center, School of Science, Walailak University, Nakhon Si Thammarat 80160, Thailand; wattana.pe@wu.ac.th; 3 Research Center for Theoretical Simulation and Applied Research in Bioscience and Sensing, Walailak University, Nakhon Si Thammarat 80160, Thailand; 4 Faculty of Animal Science and Technology, Maejo University, Chiang Mai 50290, Thailand; mju6422501001@mju.ac.th (N.V.); kridda@mju.ac.th (K.C.); 5 Department of Animal Science, Faculty of Agriculture and Environment, Savannakhet University, Savannakhet 13000, Laos; viphavanh250491@gmail.com; 6 Department of Pathology, Faculty of Medicine, Prince of Songkla University, Songkhla 90110, Thailand

**Keywords:** antibiotic resistance, *Escherichia coli*, farm to food, pathotype, public health

## Abstract

Background/Objectives: Globally, the management of infections has been complicated greatly by the rapid emergence of antibiotic resistance among bacterial pathogens, particularly *Escherichia coli* (*E. coli*). This bacterium is commonly found in a wide range of vertebrate hosts, including livestock, which can serve as important sources of specific pathogenic or multidrug-resistant strains. Cross-contamination can occur from farm to food, posing public health concerns. Methods: This cross-sectional study examined the antibiotic resistance and virulence gene profiles of *E. coli* isolated from farm animals, food processing facilities (including meat and contact surfaces), and the surrounding environment (wastewater). Results: Out of 383 samples, 230 samples (60.1%) were positive for *E. coli* (95% CI: 55.1–64.9). The prevalence rates showed significant variation across different sources, with positive rates of 72.3% (180/249) in animal sources, 33.7% (28/83) in food sources, and 43.1% (22/51) in environmental sources. Over 80% of the isolates across all sources carried the *sheA* virulence gene, which is associated with hemolytic activity in *E. coli*. Multidrug resistance (MDR) was commonly observed, with rates of 61.1% in animal samples, 57.1% in food sources, and 50.0% in environmental samples. The *E. coli* isolates exhibited high levels of antibiotic resistance, particularly to streptomycin (64.9%), ampicillin (58.0%), and tetracycline (57.6%). The most common resistance gene pattern was *tetA-strA-bla_TEM_* (22.6%). Conclusions: These findings indicate widespread occurrence of antibiotic-resistant and virulence gene-carrying *E. coli* strains across the farm-to-food continuum, underscoring the urgent need for enhanced antimicrobial stewardship and surveillance programs to mitigate transmission from food-producing animals and reduce public health complications.

## 1. Introduction

*Escherichia coli* is a Gram-negative bacterium, belonging to the family Enterobacteriaceae, and it naturally inhabits the gastrointestinal tract of warm-blooded animals. Although most *E. coli* strains are beneficial, contributing to digestion and maintaining gut microbiota balance, some strains emerge as pathogenic, posing significant health threats to animals and humans [1,2]. Pathogenic *E. coli* strains are broadly classified into two groups: (i) diarrheagenic, including adherent-invasive *E. coli* (AIEC), enteroinvasive *E. coli* (EIEC), enterotoxigenic *E. coli* (ETEC), enteropathogenic *E. coli* (EPEC), enterohemorrhagic/Shiga toxin-producing *E. coli* (EHEC/STEC), diffusely adherent *E. coli* (DAEC), and enteroaggregative *E. coli* (EAEC) [3], which cause diarrhea in both animals and humans [4]; and (ii) extraintestinal, comprising uropathogenic *E. coli* (UPEC), septicemia-associated *E. coli* (SePEC), and meningitis-associated *E. coli* (MNEC) [5], which cause extraintestinal infections such as sepsis [6].

Pathogenic *E. coli* infections are often treated with antibiotics, but the emergence of antibiotic-resistant strains, fueled by the frequent use of antibiotics, complicates effective treatment, leading to treatment failure, prolonged illness, or death. Globally, antibiotic-resistant *E. coli* poses significant public health threats due to its proficiency in acquiring and spreading resistance genes [7,8,9]. A major contributor to this resistance is the indiscriminate use of antibiotics in animal production aimed at boosting food production [10,11]. Studies indicate that antibiotics administered to animals are often incompletely metabolized, leading to environmental contamination through excretion, which further promotes antibiotic resistance [12,13]. This complicates the management of *E. coli* infections. Consequently, resistant *E. coli* strains can spread through cross-contamination from animals to food of animal origin (meat), the environment, and humans, threatening public health globally [14,15]. The World Health Organization [15] reported that in the United States, *E. coli* outbreaks primarily originated from contaminated animal-based foods (69%), water (18%), and animals (14%). Other reports have also linked human antibiotic-resistant *E. coli* to livestock, animal-based food, and the environment [16,17,18,19].

Farm animals, meat processing facilities (slaughterhouses and processing plants), and the environment are interconnected, forming essential components of the One Health framework. Consequently, bacteria from livestock can easily cross-contaminate other areas. This may escalate the resistance situation, as resistant *E. coli* in the environment can also be transmitted to other *E. coli* strains or other bacterial species in food, animals, and humans through horizontal gene transfer mechanisms, including conjugation, transformation, transduction, and vesiduction [20], posing risks to human health. Although *E. coli* may be considered part of the normal gut flora, its role as an indicator of hygiene or fecal contamination and the potential emerging antimicrobial resistance (AMR) and virulence strains as a zoonotic threat [3,5] make it significantly relevant in the modern One Health context. Despite the growing number of studies examining interconnected sources [21,22,23], most have focused on a single animal–food continuum. Comprehensive investigations that simultaneously capture antibiotic-resistant and pathogenic *E. coli* across multiple interconnected animal, food, and environmental sources within a unified framework remain limited. This knowledge gap underscores the necessity for a comprehensive, coordinated, and multi-sectoral investigation.

Therefore, this study aimed to identify and characterize antibiotic resistance and virulence determinants in *E. coli* from animals, meat processing facilities, and the surrounding environment. The data can guide or inform policies on antibiotic use in animal farms, contribute to the global reduction in diseases caused by antibiotic-resistant *E. coli* from animal sources, and improve public health.

## 2. Results

### 2.1. Prevalence of Escherichia coli from Various Sources

Prevalence of *E. coli* across the sources varied significantly (*p* < 0.001). Overall, a prevalence rate of 60.1% (230/383) (95% CI: 55.1–64.9) was recorded. The highest prevalence was observed in animal sources (72.3%), followed by environmental sources (43.1%), while food sources showed the lowest prevalence (33.7%) (Table 1). Among animal-derived samples, the overall prevalence was 72.3%, with rectal swabs yielding a prevalence of 68.9% (144/209; 95% CI: 62.2–74.7). Within this group, sheep (88.9%) and broilers (76.7%) exhibited relatively high prevalence rates (Table 1). Animal fecal samples showed the highest prevalence (90.0%; 36/40; 95% CI: 77.0–96.0), particularly in pigs (93.3%). In food sources, *E. coli* was detected in 33.7% of samples (28/83; 95% CI: 24.4–44.4), with higher prevalence in meat samples (41.9%) compared to food contact surfaces (9.5%) (Table 1). Among meat types, broiler meat showed the highest contamination (48.6%), followed by pork (40.0%) and beef (25.0%). Environmental samples, represented by wastewater, showed a prevalence of 43.1% (22/51; 95% CI: 30.5–56.7) (Table 1).

### 2.2. Virulence Gene Profiles of Escherichia coli from Various Sources

**Figure 1:**
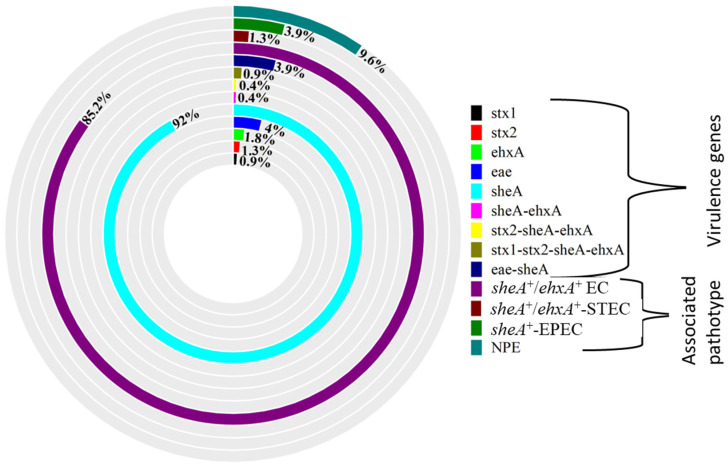
The doughnut diagram illustrates the virulence genes and pathotypes of *E. coli*. NPE: non-pathogenic *E. coli*, *sheA*^+^/*ehxA*^+^ EC: *sheA*/*ehxA*-positive *E. coli*, *sheA*^+^/*ehxA*^+^-STEC: *sheA*/*ehxA*-positive Shiga toxin-producing *E. coli*, *sheA*^+^-EPEC: *sheA*-positive enteropathogenic *E. coli*.

The *sheA* gene was the most frequently detected virulence marker among the *E. coli* isolates (92.0%), followed by the *eae* gene (4.0%), while the *stx1* gene was the least detected (0.9%) (Figure 1). Thirteen isolates harbored multiple virulence genes, with the following combinations observed: *eae*-*sheA*, *sheA*-*ehxA*, *stx1*-*stx2*-*sheA*-*ehxA*, and *stx2*-*sheA*-*ehxA* (Figure 1). Based on virulence gene profiles (Figure 1), isolates were assigned to four pathotypes: (i) non-pathogenic *E. coli* (NPE); (ii) *sheA*/*ehxA*-positive *E. coli* (*sheA*^+^/*ehxA*^+^ EC), comprising isolates harboring *sheA*, *ehxA*, or both; (iii) *sheA*/*ehxA*-positive Shiga toxin-producing *E. coli* (*sheA*^+^/*ehxA*^+^-STEC), defined by *stx2*-*sheA*-*ehxA* and *stx1*-*stx2*-*sheA*-*ehxA*; and (iv) *sheA*-positive enteropathogenic *E. coli* (*sheA*^+^-EPEC), defined by *eae*-*sheA* (Figure 1). The majority of isolates were *sheA*^+^/*ehxA*^+^ EC (85.2%), followed by NPE (9.6%), *sheA*^+^-EPEC (3.9%), and *sheA*^+^/*ehxA*^+^-STEC (1.3%) (Figure 1). No isolates harbored *bfp*, *lt*, *virF*, *hlyA*, or *eaeA* (*O157:H7*) genes.

### 2.3. Pathotype Distribution of Escherichia coli Strains from Various Sources

As shown in Table 2, the pathotype distribution varied among the sources. Based on the PCR analysis, the isolates from animal sources encompassed all identified pathotypes, with *sheA*^+^/*ehxA*^+^ EC being predominant (85.0%, 95% CI: 79.5–89.5) and non-pathogenic *E. coli* (NPE) accounting for 9.4% (95% CI: 5.5–15.1) (Table 2). Most *E. coli* isolates from food sources were *sheA*^+^/*ehxA*^+^ EC (85.7%, 95% CI: 70.5–94.3), with a small proportion classified as *sheA*^+^-EPEC (7.1%) and NPE (7.1%) (Table 2). Similarly, the majority of environmental isolates were *sheA*^+^/*ehxA*^+^ EC (86.4%, 95% CI: 68.5–95.9), while other isolates were NPE (13.6%, 95% CI: 3.1–33.6) (Table 2).

### 2.4. Antibiotic Resistance Profile of Escherichia coli

**Figure 2:**
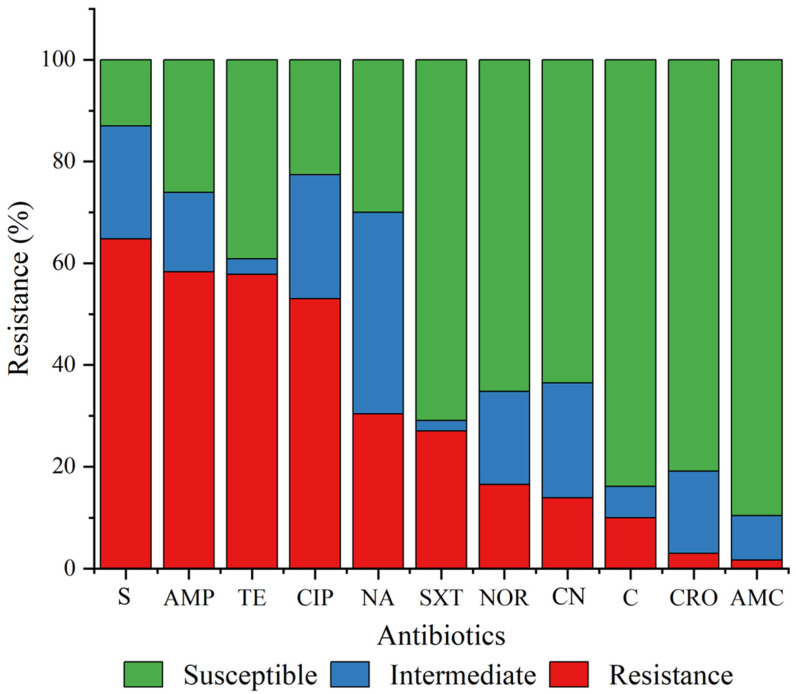
Antibiotic resistance profile of *Escherichia coli* from various sources. AMP: ampicillin, S: streptomycin, SXT: trimethoprim-sulfamethoxazole, NA: nalidixic acid, AMC: amoxicillin-clavulanic acid, TE: tetracycline, C: chloramphenicol, CN: gentamicin, CIP: ciprofloxacin, CRO: ceftriaxone, NOR: norfloxacin.

All *E. coli* isolates showed resistance to at least one of the tested antibiotics. The resistance exceeded 50.0% for streptomycin, ampicillin, tetracycline, and ciprofloxacin. The lowest resistance was observed for amoxicillin-clavulanic acid (1.7%) (Figure 2).

#### 2.4.1. Antibiotic Resistance Profiles Across Different Sources

The *E. coli* isolates exhibited varying antibiotic resistance, with the environmental isolates showing the highest resistance. However, this was not statistically significant (*p* = 0.315). Resistance rates of 28.0%, 28.6%, and 31.8% were observed among the isolates from animals, food sources, and the environment, respectively (Table 3). The isolates from animal sources were generally resistant to streptomycin (61.7%) and ciprofloxacin (60.0%). Similarly, food isolates showed high resistance to streptomycin (75.0%) and ampicillin (57.1%) (Table 3). Among the environmental isolates, 77.3% exhibited resistance to streptomycin. However, no resistance to amoxicillin-clavulanic acid was observed among the food and environmental *E. coli* isolates (Table 3).

#### 2.4.2. Multidrug Resistance Patterns in Relation to *E. coli* Pathotypes and Sources

Multidrug resistance (MDR) was observed across isolates from all sources, though the differences were not statistically significant (*p* = 0.850). Specifically, MDR was detected in 61.1% of isolates from animal sources, 57.1% from food sources, and 45.5% from environmental samples (Table 4). MDR among *sheA*^+^-EPEC isolates from animal sources ranged between 50% and 100%, while all *sheA*^+^-EPEC isolates from food sources exhibited MDR. Additionally, over half of the *sheA*^+^/*ehxA*^+^ EC isolates from all sources were found to be multidrug-resistant (Table 4). All isolates demonstrated varying levels of MDR (Table S1), with over 65% of isolates exhibiting resistance to three to four antibiotic classes (R3–4AC).

**Figure 3:**
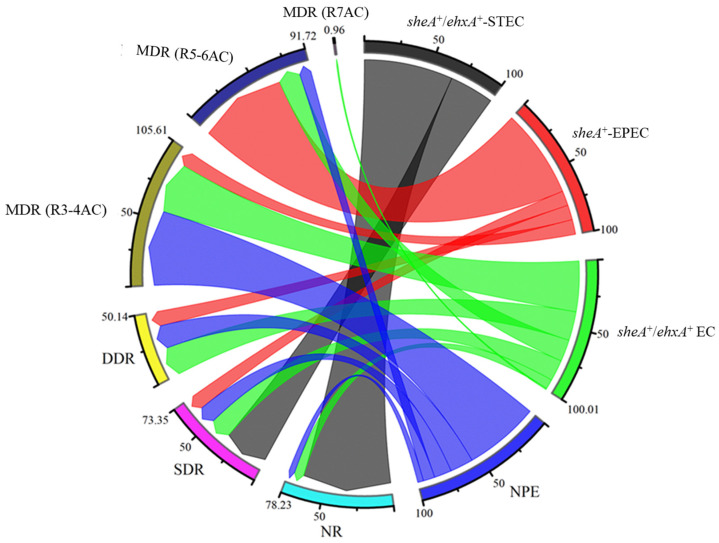
The chord diagram illustrates the association of *E. coli* pathotype with antibiotic resistance levels. NPE: non-pathogenic *E. coli*, *sheA*^+^/*ehxA*^+^ EC: *sheA*/*ehxA*-positive *E. coli*, *sheA*^+^/*ehxA*^+^-STEC: *sheA*/*ehxA*-positive Shiga toxin-producing *E. coli*, *sheA*^+^-EPEC: *sheA*-positive enteropathogenic *E. coli*. NR: non-resistance; SDR: single-drug resistance; DDR: double-drug resistance; MDR (R3–4AC): multidrug resistance (resistance to 3–4 antibiotic classes); MDR (R5–6AC): multidrug resistance (resistance to 5–6 antibiotic classes); MDR (R7AC): multidrug resistance (resistance to 7 antibiotic classes).

Resistance profiles also varied across *E. coli* pathotypes. Based on network associations, all isolates exhibited multidrug resistance (MDR), with *sheA*^+^/*ehxA*^+^ EC predominantly exhibiting resistance to three to four antibiotic classes, while some *sheA*^+^/*ehxA*^+^ EC isolates were resistant to all seven antibiotic classes tested. *sheA*^+^-EPEC pathotypes were primarily associated with resistance to five to six antibiotic classes, while NPE strains mainly showed resistance to three to four antibiotic classes. In contrast, *sheA*^+^/*ehxA*^+^-STEC pathotypes were more commonly linked to single-drug resistance (SDR) (Figure 3).

### 2.5. Antibiotic Resistance Gene Profiles of Escherichia coli from Various Sources

Various antibiotic resistance genes were identified among the isolates from different sources, with the *strA* gene recorded as the most prevalent. In total, 29 resistance gene patterns were observed, with *tetA-strA-bla_TEM_* (22.6%) showing the most common in all investigated sources (Table 5).

## 3. Discussion

This study investigated the prevalence of *E. coli* in animals, food sources (meat and contact surfaces), and the environment (wastewater) and its antibiotic resistance. The findings revealed a high prevalence of *E. coli* across all the sources investigated, with animal sources recording the highest values. This is concerning, as it may impact animal production. Also, cross-contamination could occur through contact with animals or through the consumption of contaminated meat [15]. However, sheep and broilers showed the highest prevalence of *E. coli* within the animal group. This could be attributed to variations in farm management practices across different farms. *E. coli* resides in animals, and its presence in the meat or meat processing facilities in the current study may suggest cross-contamination from animal sources, which could occur due to biosecurity or hygiene issues. Across meat types, the highest prevalence was recorded in broilers meat, possibly reflecting differences in farming practices and meat processing conditions. However, the prevalence reported in this study is lower than that previously reported in food from animal sources (meat: 72%) in Thailand [24], in broilers in the UAE (82.2%) [25], and in the farm-to-food continuum in Canada (95.6–97.4%) [21]. Methods of transportation of animals from farms to slaughterhouses, lairage conditions (overcrowding in slaughterhouse pens and fecal shedding, which leads to fecal buildup), slaughter processes, and poor biosecurity measures in slaughterhouses could facilitate the cross-transmission of bacteria from animals or meat to humans [20,26], posing a potential threat to public health.

Although *E. coli* is generally regarded as part of the normal flora in the gastrointestinal tract of animals, the emerging virulent trains, particularly in food-producing animals, pose a significant health concern [3,5]. These strains may acquire and exchange genetic elements in the environment and across different sources, which can influence their genetic profile. PCR analysis in the present study indicated a relatively high prevalence of *E. coli* isolates carrying hemolysin-associated genes (*sheA* and *ehxA*), with the *sheA* gene detected more frequently than *ehxA*, and with no detection of *hlyA*. This pattern is consistent with findings reported by Lorenz et al. [27], who observed a high occurrence of *sheA* (93.8%) and *ehxA* (69.2%) genes in food, animals, and humans in the USA, with the *hlyA* gene rarely recovered. The presence of the *sheA* gene has been linked to hemolytic potential in some *E. coli* strains [28,29]; however, phenotypic confirmation (e.g., hemolysis assay) was not performed in this study, and therefore functional expression cannot be inferred. This demands further investigation. Other virulence-associated genes, including *eae*, *stx1*, and *stx2*, were detected at lower frequencies. Their presence in *E. coli* isolates from livestock and meat may be of interest from an epidemiological perspective, as these genes may serve as indicators of genetic elements associated with strains of clinical relevance [30], and in some cases they were observed alongside the *sheA* gene. This is consistent with other studies that reported multiple virulence genes in *E. coli* [31,32]. This could potentially increase the pathogenicity of these strains; however, this remains uncertain without further functional characterization. The similarity of virulence genes recovered from the sources in the current study suggests that these *E. coli* strains may be interrelated or originated from a common source, indicating a potential for zoonotic transmission from animals to meat, and ultimately to humans. While some strains appeared to have no virulence genes, it is worth noting that they could harbor other virulence genes that were not identified in the current study. A further limitation is the use of a single isolate per sample, which may not capture within-sample diversity and could lead to underestimation of gene prevalence.

The emergence of antibiotic resistance in *E. coli* has made it a significant One Health pathogen worldwide. The current study assessed antibiotic-resistant *E. coli* from animals, food sources (meat and contact surfaces), and the environment (wastewater), which revealed a high prevalence (28–31.8%), particularly against streptomycin, ampicillin, and tetracycline, in all sources. Interestingly, resistance to ciprofloxacin was higher than that observed for nalidixic acid, which contrasts with the typical stepwise pattern of quinolone resistance. This discrepancy may be explained by the presence of additional resistance mechanisms, such as plasmid-mediated quinolone resistance determinants or efflux pumps, as well as selective pressure from the use of fluoroquinolones in animal production [33]. Furthermore, specific mutation patterns in quinolone resistance-determining regions (QRDRs) may differentially affect susceptibility to first- and second-generation quinolones [34]. Similar previous studies in Thailand have reported high streptomycin- and tetracycline-resistant *E. coli* [35,36]. This could be attributed to these antibiotics being widely used for animal production [10,11,35]. This induces antibiotic resistance over time due to selective pressure. However, the resistance reported in the current study is lower than that reported in previous studies, including those conducted in the farm-to-food continuum in South Africa (ampicillin: 100%, tetracycline: 100%) [22] and in broilers in the UAE (ampicillin: 100%, tetracycline: 72.6%, and ciprofloxacin: 88.4%) [25]. All isolates from food sources (meat and contact surfaces) exhibited susceptibility to amoxicillin-clavulanic acid. This conforms with Abou-Jaoudeh et al. [37], who reported low (13.9%) amoxicillin-clavulanic acid-resistant *E. coli* in chicken meat in Lebanon. This broadens the available treatment options for infections caused by antibiotic-resistant *E. coli* from animal sources. The resistance recorded in animals was lower than in previous reports in livestock (70–78.3%) [35,38]. This could be attributed to differences in farm management practices, such as variations in antibiotic use across different jurisdictions. High antibiotic resistance was also recorded in the environmental (wastewater) isolates. Wastewater can harbor diverse microbes, including antibiotic-resistant *E. coli* strains. A previous study in Ireland identified wastewater as a reservoir for carbapenem-resistant bacteria [39]. The current results indicate similar resistance characteristics exhibited by isolates from different sources, highlighting their interrelatedness and a potential risk of cross-contamination among animals, food (meat and contact surfaces), and the environment, thereby posing a public health threat.

Multidrug resistance (MDR) is usually associated with bacteria harboring multiple resistance genes that encode for different antibiotic resistance, an increase in gene expression, efflux pumps of antibiotics [40], or with other non-genetic factors. Animals and animal feces recorded the highest MDR in the current study. This could be attributed to the indiscriminate use of antibiotics in animal production [12,13]. This could induce antibiotic resistance in the environment by selective pressure and its subsequent potential effect on animal production and the ripple effect on human health. The MDR recorded from the animals in this study is higher than the MDR (23.9%) reported by Prapasawat and Intarapuk [35] in Thailand. However, a similar study along the pork production chain in central Thailand reported a higher prevalence of MDR (87.3%) [23]. This is directly linked to variations in antibiotic application for animal production in different jurisdictions and farms [41]. The current findings may negatively impact human health through direct contact with farm animals harboring these MDR *E. coli* or via the animal-based food chain. The MDR in the food sources is higher than the multidrug resistance (49.23%) reported in meat in Bangladesh [42]. This discrepancy could also be ascribed to antibiotic usage at farms at different locations. However, the major concern is the high level of MDR and resistance to five to six antibiotic classes recorded among the supposedly non-pathogenic *E. coli* strains across all sources, resistance to seven antibiotic classes among the *sheA*^+^/*ehxA*^+^ EC, and resistance to five to six antibiotic classes among the *sheA*^+^-EPEC pathotypes in the current study. This implies that the treatment options for bacterial strains from these sources are gradually diminishing. This, therefore, demands public sensitization, including veterinary services, and farmers’ awareness of the consequences of the indiscriminate use of antibiotics for animal farming, as this could negatively impact animal and human health.

The continuous application of antibiotics exerts selective pressure on bacteria, driving the development of various resistance mechanisms over time, including genotypic resistance. This study assessed the genotypic resistance of *E. coli* isolates. The highest resistance gene pattern observed in all the sources was *tetA-strA-bla_TEM_*_._ These genes encode resistance to tetracyclines, aminoglycosides (streptomycin), and penicillin (e.g., ampicillin) or cephalosporins (ceftriaxone). This could be attributed to the widespread use of these antibiotics in animal production [35], which exerts selective pressure for the expression of these resistance genes in the bacteria. This may have contributed to the increased phenotypic resistance observed in this study. For instance, the *bla_TEM_* gene in *E. coli* encodes the enzyme beta-lactamase that hydrolyzes the beta-lactam rings in beta-lactam antibiotics [43,44]. The *tetA* gene encodes an inducible efflux pump where a complex of tetracycline and divalent metallic ions is translocated out of the cell, reducing the concentration of tetracycline in the bacterial cell and protecting the ribosomes from the action of tetracycline antibiotics. In contrast, the *strA* gene mediates enzymatic modification of aminoglycosides. Consequently, antibiotics such as aminoglycosides (e.g., streptomycin) become ineffective [43]. The presence of these resistance gene patterns demonstrates the mechanisms by which *E. coli* from these current studied sources resist these antibiotics. This reduces treatment options and poses the risk of these genes being transferred to other non-resistant *E. coli*. This could have significant public health implications, demanding strict measures regarding the application of these antibiotics for animal production, as resistant *E. coli* in the environment can transmit resistance genes to other *E. coli* (through vertical gene transmission) or to other bacteria in food, animals, or humans (horizontal gene transfer) (through conjugation, transformation, transduction, and vesiduction) [20].

## 4. Materials and Methods

### 4.1. Interconnected Sampling Sites, Study Design, and Sample Collection

The research is a cross-sectional study in which a multistage random sampling technique was used to collect samples from designated sites to analyze the prevalence and distribution of antibiotic resistance and virulence genes in *E. coli* along the farm-to-fork continuum. Permission was obtained from the management of each area prior to sampling. Sampling was done from July 2023 to December 2023.

A total of 383 samples were randomly collected from farm animals, food processing facilities (slaughterhouses or meat processing plants), and the surrounding environments (wastewaters). To ensure the interconnectedness of these sectors, only animal farms linked (closed proximity and farms that supply animals to the selected processing facilities) to meat processing facilities were selected in this study. Likewise, only wastewater sources that were traceable to meat processing facilities under the investigation were included in the study.

A total of 249 samples from animal sources were randomly collected from farm animals. Rectal swabs were obtained from chicken, ducks, turkeys, guinea fowls, goats, and sheep using sterile cotton swabs gently inserted into the cloaca or anus. Fecal samples were collected from pigs and cattle. All samples were aseptically collected from farm animals in Bangkok, Chiang Mai, and Nakhon Ratchasima, Thailand, and each was individually stored in a sterile zip-lock plastic bag.

A total of 83 food-related samples were randomly collected from meat processing facilities in Bangkok, Prachinburi, and Saraburi for analysis. These included chicken, pork, and beef meat samples (approximately 100 g each) and surface swabs. Surface samples were obtained by swabbing contact areas (approximately 10 cm^2^ per surface) with sterile cotton swabs moistened with sterile saline. The sampling sites within the processing environments included utensils (chopping boards, weighing scales, pans, and butcher knives) and tables in slaughterhouses. All samples were aseptically collected and individually stored in sterile zip-lock bags. In addition, a total of 51 wastewater samples (~500 mL) linked to the meat processing facilities were aseptically collected in sterile bottles.

All samples were clearly labeled, stored in an ice box containing ice packs, and transported promptly to the laboratory for bacteriological analysis. These samples were processed in the Laboratory of Bacteriophage and Food Safety, Department of Biotechnology, Faculty of Agro-Industry, Kasetsart University, Thailand.

### 4.2. Sample Preparation and Isolation of Escherichia coli

Sample preparation and isolation followed an approach proposed by Feng et al. [45] with some modifications. Briefly, swab samples were immersed in 9 mL of buffered peptone water (BPW) (#CM1040, OXOID Ltd., Basingstoke, UK) in sterile test tubes. For solid samples (meat and animal feces/droppings), 25 g of each sample was transferred into sterile zip-lock bags containing 225 mL of BPW. Liquid samples (wastewater) were prepared by inoculating 25 mL of each homogenized sample into 225 mL of BPW in sterile zip-lock bags. All samples were homogenized and incubated at 37 °C for 24 h. Subsequently, a loopful of the enriched mixture was streaked onto Eosin Methylene Blue (EMB) agar plates (#M022, Himedia Laboratories Pvt. Ltd., Nagpur, Maharashtra, India) and incubated at 37 °C for 24 h. Colonies exhibiting a characteristic metallic green sheen on EMB were considered presumptive *E. coli*. Selected colonies were re-streaked on nutrient agar and incubated at 37 °C for an additional 24 h to obtain pure cultures for further biochemical confirmation. *E. coli* ATCC^®^ 25922 (American Type Culture Collection, Manassas, VA, USA) was used as a positive control strain for the *E. coli* isolation.

### 4.3. Biochemical Identification of Escherichia coli

Biochemical confirmation of the presumptive *E. coli* followed Feng et al. [45] with slight modifications: The presumptive *E. coli* isolates were confirmed using the Triple Sugar Ion (TSI) agar test (#CM0277, OXOID Ltd., Basingstoke, UK), indole test, the O-Nitrophenyl-β-D-Galactopyranoside (ONPG) test as described by Feng et al. [45], and further confirmed using the LIA agar test (#M377, Himedia Laboratories Pvt. Ltd., Maharashtra, India) and citrate test (#M099, Himedia Laboratories Pvt. Ltd., Maharashtra, India). *E. coli* ATCC^®^ 25922 was used as a positive control strain for the biochemical confirmations. After this, *E. coli* was further confirmed through PCR according to an approach described by Azimi et al. [46], using specific primer for the *uidA* gene (Table S2; Figure S1). The *E. coli* O157:H7 strain from Wiriyaprom et al. [47] was used as a positive control, while the PCR reaction without a DNA sample served as a negative control.

### 4.4. Antibiotic Susceptibility Testing of Escherichia coli

Antibiotic susceptibility was tested using the Kirby–Bauer disk diffusion method following the Laboratory Standards Institute (CLSI) guidelines. Mueller–Hinton agar (CM0337, OXOID Ltd., Basingstoke, UK) plates were inoculated with 24 h tryptic soy broth (#M011, Himedia Laboratories Pvt. Ltd., Maharashtra, India), *E. coli* cultures adjusted to a 0.5 McFarland standard, and antibiotic disks applied and incubated at 37 °C for 18 h [48,49]. Antibiotics, including ampicillin (10 µg), streptomycin (10 µg), trimethoprim-sulphamethoxazole (25 µg), nalidixic acid (30 µg), amoxicillin-clavulanic acid (30 µg), tetracycline (30 µg), chloramphenicol (30 µg), gentamicin (10 µg), ciprofloxacin (5 µg), ceftriaxone (30 µg), and norfloxacin (10 µg), were used. Besides being recommended for *E. coli* in the CLSI guidelines [49], these antibiotics are commonly used in animal production [31]; therefore, they were carefully selected for this study. After incubation, the inhibition zones were recorded and interpreted using the CLSI breakpoints [50]. *E. coli* ATCC^®^ 25922 was used as a quality control strain in this study. Isolates exhibiting resistance to one and two antibiotic classes were identified as single-drug resistant (SDR) and double-drug resistant (DDR), respectively, while isolates exhibiting resistance to three or more antibiotic classes were identified as multidrug-resistant strains.

### 4.5. Determination of Virulence and Antibiotic Resistance Genes Among Escherichia coli

#### 4.5.1. DNA Extraction

DNA of the *E. coli* isolates was extracted using a genomic DNA Prep kit (#GD 141-050, BIOFACT^TM^, BIOFACT Co., Ltd., Daejeon, Republic of Korea) for PCR analysis. The DNA was suspended in 100 µL of DNA hydration buffer, and 2 µL of the eluted DNA was used as the template for each PCR reaction.

#### 4.5.2. Detection of Virulence Genes

Specific primers for representative virulence genes (Table S2) were used for the PCR following Azimi et al. [46] with slight modifications. Each 25 µL PCR reaction contained a PCR mix (2.5 µL of 10× PCR buffer, 2 µL of 2.5 mM dNTP, and 0.2 µL of 5U Taq polymerase) (#W1301, Wizbiosolution Inc., Seongnam, Gyeonggi-do, Republic of Korea), 16.3 µL of nucleotide-free deionized water, 2 µL of the DNA template, and 1 µL of 0.25 µM of each primer (forward and reverse). PCR was run using a thermocycler (BIORAD T100^TM^ Thermal cycler, Bio-Rad Laboratories Ltd., Hercules, CA, USA) with gene-specific cycling conditions as follows: initial denaturation for 15 min at 95 °C for *sheA* and *ehxA* genes only [27,51], 5 min at 95 °C for all the remaining genes, followed by 25 cycles for *sheA* and *ehxA* genes only, 35 cycles for all the remaining genes, each cycle consisting of denaturation for 5 min at 94–95 °C, annealing for 30 s at 54–60 °C, extension for 1 min at 72 °C, and a final extension step for 5 min at 72 °C. The *E. coli* (O157:H7) and F1.1 strains from Wiriyaprom et al. [47] were used as quality control strains in this study, while a PCR reaction without a DNA sample served as a negative control. All experiments were done in triplicate.

#### 4.5.3. Detection of Antibiotic Resistance Genes

The detection of representative antibiotic resistance genes for aminoglycosides (*strA*, *aac3-(IV)*), tetracycline (*tetA*), beta-lactam (*bla_TEM_*), sulfonamides (*sul1*, *sul2*, *sul3*), and phenicol (*catI*, *catII,* and *floR*) was performed through PCR [52,53,54,55,56,57] with slight modifications using specific primers (Table S3). The PCR mixture as described above was used. The PCR was run with a thermocycler (BIORAD T100^TM^ Thermal cycler, Bio-Rad Laboratories Ltd., CA, USA) using the following conditions: initial denaturation for 5 min at 94–95 °C, followed by 30–35 cycles, each consisting of denaturation at 98 °C for 1 min for *sul2* and *sul3* genes only, 30–60 s at 94–95 °C for all the remaining genes, annealing for 30–60 s at 50–62.5 °C, extension for 1 min at 72 °C, and a final extension step for 5 min at 72 °C. Multidrug-resistant clinical *E. coli* strain (EC3.18) served as a positive control for resistance gene detection, while a PCR reaction without a DNA sample served as a negative control. All experiments were done in triplicate.

#### 4.5.4. Visualization of Amplicons

All the amplified samples (amplicons) were analyzed through gel electrophoresis with a 1.5% agarose gel (#PC0701, Vivantis Technology Sdn Bhd, Subang Jaya, Selangor, Malaysia) pre-stained with 1% SYBR green (SYBR^TM^ Green I nucleic acid gel, Invitrogen^TM^ Thermo Fisher Scientific^TM^, Waltham, MA, USA), and the bands were observed using a gel documentation system (iBright^TM^ CL1500 imaging system, Invitrogen^TM^ Thermo Fisher Scientific^TM^, Waltham, MA, USA). It was then compared with a standard-sized molecular marker (DNA ladder) (#NL1407, Vivantis Technology Sdn Bhd, Subang Jaya, Selangor, Malaysia) of 100 bp increments.

### 4.6. Statistical and Data Analysis

The data were scrutinized for completeness and analyzed using SPSS (IBM^®^ SPSS^®^ Statistic version 25, SPSS Inc., Chicago, IL, USA). The chi-square test was used to assess significant differences at the 95% confidence level, and a *p*-value of ≤0.05 was considered statistically significant. The graphs were generated using ORIGIN PRO^®^ v.2024b Graphing and Analysis (OriginLab Corporation, Northampton, MA, USA).

## 5. Conclusions

A high prevalence of *E. coli* was observed across all the sources, with over 80% of the isolates identified as *sheA*^+^/*ehxA*^+^ EC. The *E. coli* isolates exhibited significant antibiotic resistance, with the highest resistance recorded for streptomycin, ampicillin, and tetracycline. Additionally, most of the *E. coli* isolates were multidrug-resistant. The most prevalent antibiotic resistance gene pattern was *tetA-strA-bla_TEM_*. Alarmingly, both virulence gene-carrying *E. coli* and NPE strains displayed MDR, often carrying multiple resistance genes. These constitute a possibility for zoonotic infection that could lead to complications, demanding an urgent need for effective antimicrobial stewardship policies to regulate the use of antibiotics in animal production, and robust systems to reduce resistance and virulence genes of *E. coli* from farm to fork. Future works should focus on the whole genome sequencing of these *E. coli* isolates.

## Figures and Tables

**Table 1:** Prevalence of *Escherichia coli* among different sources.

Sources	Sample Types	Category	Positive Sample/Total Samples	% Prevalence	95% CI
Animals	Rectal swab	Chicken	92/120	76.7	69.1–84.3
		Ducks	32/45	71.1	57.8–84.4
		Turkey	2/10	20.0	0.0–44.8
		Guinea fowls	3/15	20.0	0.0–40.2
		Goat	7/10	70.0	41.6–98.4
		Sheep	8/9	88.9	68.4–100.0
		Subtotal	144/209	68.9	62.2–74.7
	Animal feces	Pigs	28/30	93.3	84.4–100.0
		Cattle	8/10	80.0	55.2–100.0
		Subtotal	36/40	90.0	77.0–96.0
	Total	-	180/249	72.3	66.4–77.4
Foods	Meat	Chicken meat	17/35	48.6	32.0–65.1
		Pork	6/15	40.0	15.2–64.8
		Beef	3/12	25.0	0.5–49.5
		Subtotal	26/62	41.9	30.5–54.2
	Food contact surface (swab samples)	Utensils	2/13	15.4	0.0–35.0
		Table	0/8	0.0	0.0–0.0
		Subtotal	2/21	9.5	2.7–29.3
	Total	-	28/83	33.7	24.4–44.4
Environment	Wastewater	-	22/51	43.1	30.5–56.7
Overall			230/383	60.1	

CI: confidence interval.

**Table 2:** Distribution of *Escherichia coli* pathotypes among different sources.

Sources	Sample Types	N	Pathotypes (N (%), [95% CI])			
			*sheA*^+^/*ehxA*^+^ EC	*sheA*^+^/*ehxA*^+^-STEC	*sheA*^+^-EPEC	NPE
Animals	Rectal swab	144	127 (88.2), [82.1–92.6]	3 (2.1), [0.7–4.9]	3 (2.1), [0.7–4.9]	11 (7.6), [3.9–13.1]
	Animal feces	36	26 (72.2), [56.0–84.8]	0 (0.0), [0.0–8.3]	4 (11.1), [3.9–25.6]	6 (16.7), [6.9–33.7]
	Total	180	153 (85.0), [79.5–89.5]	3 (1.6), [0.4–4.5]	7 (3.9), [1.8–7.8]	17 (9.4), [5.5–15.1]
Foods	Meat	26	23 (88.5), [73.0–96.2]	0 (0.0), [0.0–11.4]	2 (7.6), [1.1–24.1]	1 (3.9), [0.1–20.4]
	FCS	2	1 (50.0), [11.5–88.5]	0 (0.0), [0.0–60.2]	0 (0.0), [0.0–60.2]	1 (50.0), [11.5–88.5]
	Total	28	24 (85.7), [70.5–94.3]	0 (0.0), [0.0–9.9]	2 (7.1), [1.6–21.2]	2 (7.1), [1.6–21.2]
Environment	Wastewater	22	19 (86.4), [68.5–95.9]	0 (0.0), [0.0–13.2]	0 (0.0), [0.0–13.2]	3 (13.6), [3.1–33.6]

CI: confidence interval, NPE: non-pathogenic *E. coli*, *sheA*^+^/*ehxA*^+^ EC: *sheA*/*ehxA*-positive *E. coli*, *sheA*^+^/*ehxA*^+^-STEC: *sheA*/*ehxA*-positive Shiga toxin-producing *E. coli*, *sheA*^+^-EPEC: *sheA*-positive enteropathogenic *E. coli*. FCS: food contact surfaces.

**Table 3:** Antibiotic resistance profile of *Escherichia coli* from various sources.

Antibiotics	% Resistance Profile in Various Sources								
	Animals (180)			Food Source (28)			Environment (22)		
	R	I	S	R	I	S	R	I	S
S	61.7	23.9	14.4	75.0	14.3	10.7	77.3	18.2	4.5
AMP	59.4	18.3	22.2	57.1	0.0	42.9	50.0	13.6	36.4
TE	60.0	3.3	36.7	53.6	3.6	42.9	54.5	0.0	45.5
CIP	52.2	23.9	23.9	53.6	21.4	25.0	59.1	31.8	9.1
NA	28.9	40.6	30.6	35.7	28.6	35.7	36.4	45.5	18.1
SXT	27.2	2.8	70.0	25.0	0.0	75.0	27.3	0.0	72.7
NOR	17.8	17.8	64.4	3.6	28.6	67.8	22.7	9.1	68.2
CN	11.7	22.8	65.6	25.0	17.9	57.1	18.2	27.3	54.5
C	10.0	6.7	83.3	7.1	3.6	89.3	13.6	4.6	81.8
CRO	3.0	16.1	81.1	0.0	17.9	82.1	9.1	13.6	77.3
AMC	2.0	10.6	87.4	0.0	0.0	100.0	0.0	4.5	95.5
Overall	28.1	23.7	48.3	28.6	18.8	52.7	31.8	20.5	47.7

R: resistance, I: intermediate, S: susceptible, AMP: ampicillin, S: streptomycin, SXT: trimethoprim-sulfamethoxazole, NA: nalidixic acid, AMC: amoxicillin-clavulanic acid, TE: tetracycline, C: chloramphenicol, CN: gentamicin, CIP: ciprofloxacin, CRO: ceftriaxone, NOR: norfloxacin.

**Table 4:** Prevalence of multidrug-resistant *Escherichia coli* from various sources.

Sources	Sample Types	MDR Isolate (N)	Multidrug Resistance (%)			
			*sheA*^+^/*ehxA*^+^ EC	*sheA*^+^/*ehxA*^+^-STEC	*sheA*^+^-EPEC	NPE
Animals	Rectal swab	85/144 (59.0)	76/127 (59.8)	0/3 (0.0)	2/3 (66.7)	7/11 (63.6)
	Animal feces	24/36 (66.7)	18/26 (69.2)	0/0 (0.0)	3/4 (75.0)	3/6 (50.0)
	Total	110/180 (61.1)	95/153 (62.1)	0/3 (0.0)	5/7 (71.4)	10/17 (58.8)
Foods	Meat	15/26 (57.7)	13/23 (56.5)	0 (0.0)	2/2 (100.0)	0/1 (0.0)
	FCS	1/2 (50.0)	0/1 (0.0)	0/1 (0.0)	0/1 (0.0)	1/1 (100.0)
	Total	16/28 (57.1)	13/24 (54.2)	0 (0.0)	2/2 (100.0)	1/2 (50.0)
Environment	Wastewater	10/22 (45.5)	9/19 (47.4)	0/0 (0.0)	0/0 (0.0)	1/3 (33.3)

NPE: non-pathogenic *E. coli*, *sheA*^+^/*ehxA*^+^ EC: *sheA*/*ehxA*-positive *E. coli*, *sheA*^+^/*ehxA*^+^-STEC: *sheA*/*ehxA*-positive Shiga toxin-producing *E. coli*, *sheA*^+^-EPEC: *sheA*-positive enteropathogenic *E. coli*. FCS: Food contact surfaces.

**Table 5:** Antibiotic-resistant gene profiles of *Escherichia coli*.

ABR Profiles	ABR Gene Profiles	Frequency (%)
RG1	*bla_TEM_*	10 (4.4)
RG2	*strA*	41 (17.8)
RG3	*strA-bla_TEM_*	20 (8.7)
RG4	*sul1-strA*	2 (0.9)
RG5	*sul1-strA-bla_TEM_*	1 (0.4)
RG6	*sul2-strA-bla_TEM_*	1 (0.4)
RG7	*sul2-strA-bla_TEM_-catI*	1 (0.4)
RG8	*sul3-bla_TEM_*	1 (0.4)
RG9	*tetA*	3 (1.3)
RG10	*tetA-bla_TEM_*	12 (5.2)
RG11	*tetA-bla_TEM_-floR*	3 (1.3)
RG12	*tetA-strA*	14 (6.1)
RG13	*tetA-strA-bla_TEM_*	52 (22.6)
RG14	*tetA-strA-bla_TEM_-catII*	7 (3.0)
RG15	*tetA-strA-bla_TEM_-floR*	4 (1.7)
RG16	*tetA-strA-catII*	1 (0.4)
RG17	*tetA-sul1-bla_TEM_*	2 (0.9)
RG18	*tetA-sul1-strA-bla_TEM_*	2 (0.9)
RG19	*tetA-sul1-strA*	1 (0.4)
RG20	*tetA-sul1-sul2-strA-bla_TEM_*	3 (1.3)
RG21	*tetA-sul1-sul2-sul3-strA-bla_TEM_*	1 (0.4)
RG22	*tetA-sul2-strA*	2 (0.9)
RG23	*tetA-sul2-strA-bla_TEM_*	6 (2.6)
RG24	*tetA-sul2-strA-bla_TEM_-catI*	1 (0.4)
RG25	*tetA-sul2-strA-bla_TEM_-floR*	3 (1.3)
RG26	*tetA-sul2-sul3-bla_TEM_*	1 (0.4)
RG27	*tetA-sul3-bla_TEM_*	5 (2.2)
RG28	*tetA-sul3-strA*	1 (0.4)
RG29	*tetA-sul3-strA-bla_TEM_*	13 (5.7)

*tetA*: tetracycline resistance gene, *bla_TEM_*: beta-lactamase gene; *strA*: streptomycin resistance gene, *sul1*, *sul2*, *sul3*: sulfonamide resistance genes, *catI*: chloramphenicol acetyltransferase gene, *floR*: phenicol resistance gene.

## Data Availability

All data generated or analyzed during this study are included in this published article and its Supplementary Materials files.

## Supplementary Materials

The following supporting information can be downloaded at: https://www.mdpi.com/article/10.3390/antibiotics15050455/s1, Figure S1: Gel image of *uidA* gene for *E. coli* identification; Table S1: Multidrug resistance patterns of *E. coli* from various sources; Table S2: Primer lists for virulence genes; Table S3: Primer lists for antibiotic-resistant genes.
