# Supplementary material for: Widespread Multidrug Resistance and Virulence Determinants in Escherichia coli Across the Interconnected Farm-to-Food Continuum
Source: Antibiotics (Basel). 2026 Apr 30;15(5):455. doi: 10.3390/antibiotics15050455 (PMC13203569; doi:10.3390/antibiotics15050455)
Supplement: Supplementary file 1 [file antibiotics-15-00455-s001.zip › antibiotics-4235015-supplementary.pdf]

Table S1. Multidrug resistance patterns of *E. coli* from various sources.

Profile	Multidrug resistance	Aminoglycoside	Quinolones/ Fluoroquinolones	Penicillin	Cephalosporin	Sulfonamides	Phenicol	Tetracyclines	Resistant type
AR1	AMP-S-CIP	S	CIP	AMP					MDR (R3AC)
AR2	AMP-S-CIP-NOR	S	CIP,NOR	AMP					MDR (R3AC)
AR3	AMP-S-NA	S	NA	AMP					MDR (R3AC)
AR4	AMP-SXT-CIP		CIP	AMP		SXT			MDR (R3AC)
AR5	AMP-S-TE	S		AMP				TE	MDR (R3AC)
AR6	AMP-S-TE-CN	S,CN		AMP				TE	MDR (R3AC)
AR7	AMP-SXT-TE			AMP		SXT		TE	MDR (R3AC)
AR8	AMP-TE-CIP		CIP	AMP				TE	MDR (R3AC)
AR9	S-TE-CIP	S	CIP					TE	MDR (R3AC)
AR10	AMP-NA-C-CIP		NA,CIP	AMP			C		MDR (R3AC)
AR11	AMP-NA-TE-CIP		NA,CIP	AMP				TE	MDR (R3AC)
AR12	AMP-S-AMC-TE	S		AMP,AMC				TE	MDR (R3AC)
AR13	AMP-S-NA-CIP-NOR	S	NA,CIP,NOR	AMP					MDR (R3AC)
AR14	AMP-S-NA-CN-CIP-NOR	S,CN	NA,CIP,NOR	AMP					MDR (R3AC)
AR15	AMP-TE-CIP-NOR		CIP,NOR	AMP				TE	MDR (R3AC)
AR16	NA-TE-CN-CIP-NOR	CN	NA,CIP,NOR					TE	MDR (R3AC)
AR17	S-NA-TE-CIP-NOR	S	NA,CIP,NOR					TE	MDR (R3AC)
AR18	SXT-NA-TE-CIP		NA,CIP			SXT		TE	MDR (R3AC)
AR19	AMP-TE-CIP-CRO		CIP	AMP	CRO			TE	MDR (R4AC)
AR20	AMP-NA-TE-CN-CIP	CN	NA,CIP	AMP				TE	MDR (R4AC)
AR21	AMP-S-NA-TE	S	NA	AMP				TE	MDR (R4AC)
AR22	AMP-S-SXT-CN-CIP	S,CN	CIP	AMP		SXT			MDR (R4AC)
AR23	AMP-S-SXT-NA	S	NA	AMP		SXT			MDR (R4AC)

Profile	Multidrug resistance	Aminoglycoside	Quinolones/ Fluoroquinolones	Penicillin	Cephalosporin	Sulfonamides	Phenicol	Tetracyclines	Resistant type
AR24	AMP-S-SXT-TE	S		AMP		SXT		TE	MDR (R4AC)
AR25	AMP-S-SXT-TE-CN	S,CN		AMP		SXT		TE	MDR (R4AC)
AR26	AMP-S-TE-C	S		AMP			C	TE	MDR (R4AC)
AR27	AMP-S-TE-CIP	S	CIP	AMP				TE	MDR (R4AC)
AR28	AMP-S-TE-CN-CIP	S,CN	CIP	AMP				TE	MDR (R4AC)
AR29	AMP-SXT-NA-TE-CIP		NA,CIP	AMP		SXT		TE	MDR (R4AC)
AR30	AMP-SXT-TE-CIP		CIP	AMP		SXT		TE	MDR (R4AC)
AR31	S-SXT-TE-C	S				SXT	C	TE	MDR (R4AC)
AR32	AMP-TE-CN-CIP	CN	CIP	AMP				TE	MDR (R4AC)
AR33	AMP-S-NA-TE-CIP	S	NA,CIP	AMP				TE	MDR (R4AC)
AR34	AMP-S-AMC-TE-CIP	S	CIP	AMP,AMC				TE	MDR (R4AC)
AR35	AMP-S-NA-TE-CIP-NOR	S	NA,CIP,NOR	AMP				TE	MDR (R4AC)
AR36	AMP-S-NA-TE-CN-CIP	S,CN	NA,CIP	AMP				TE	MDR (R4AC)
AR37	AMP-S-SXT-NA-CIP	S	NA,CIP	AMP		SXT			MDR (R4AC)
AR38	AMP-S-NA-AMC-TE-CIP-NOR	S	NA,CIP,NOR	AMP,AMC				TE	MDR (R4AC)
AR39	AMP-SXT-NA-TE-CIP-NOR		NA,CIP,NOR	AMP		SXT		TE	MDR (R4AC)
AR40	AMP-S-SXT-TE-C	S		AMP		SXT	C	TE	MDR (R5AC)
AR41	AMP-S-NA-TE-C	S	NA	AMP			C	TE	MDR (R5AC)
AR42	AMP-S-SXT-NA-TE-CN	S,CN	NA	AMP		SXT		TE	MDR (R5AC)
AR43	AMP-S-SXT-TE-CIP	S	CIP	AMP		SXT		TE	MDR (R5AC)
AR44	AMP-S-SXT-TE-CN-CIP	S,CN	CIP	AMP		SXT		TE	MDR (R5AC)
AR45	AMP-S-TE-C-CIP	S	CIP	AMP			C	TE	MDR (R5AC)
AR46	AMP-SXT-TE-C-CIP		CIP	AMP		SXT	C	TE	MDR (R5AC)
AR47	AMP-SXT-NA-TE-CIP-CRO		NA,CIP	AMP	CRO	SXT		TE	MDR (R5AC)
AR48	AMP-S-NA-TE-C-CIP-NOR	S	NA,CIP,NOR	AMP			C	TE	MDR (R5AC)

Profile	Multidrug resistance	Aminoglycoside	Quinolones/ Fluoroquinolones	Penicillin	Cephalosporin	Sulfonamides	Phenicol	Tetracyclines	Resistant type
AR49	AMP-S-SXT-NA-TE-CIP	S	NA,CIP	AMP		SXT		TE	MDR (R5AC)
AR50	AMP-S-SXT-NA-TE-CIP-NOR	S	NA,CIP,NOR	AMP		SXT		TE	MDR (R5AC)
AR51	AMP-S-SXT-NA-TE-CN-CIP	S,CN	NA,CIP	AMP		SXT		TE	MDR (R5AC)
AR52	S-SXT-NA-TE-C-CN-CIP-NOR	S,CN	NA,CIP,NOR			SXT	C	TE	MDR (R5AC)
AR53	AMP-S-SXT-NA-AMC-TE-CIP-NOR	S	NA,CIP,NOR	AMP,AMC		SXT		TE	MDR (R5AC)
AR54	AMP-S-SXT-NA-TE-C	S	NA	AMP		SXT	C	TE	MDR (R6AC)
AR55	AMP-S-SXT-NA-TE-CN-CIP-CRO-NOR	S,CN	NA,CIP,NOR	AMP	CRO	SXT		TE	MDR (R6AC)
AR56	AMP-S-SXT-NA-TE-C-CIP	S	NA,CIP	AMP		SXT	C	TE	MDR (R6AC)
AR57	AMP-S-SXT-NA-TE-C-CIP-NOR	S	NA,CIP,NOR	AMP		SXT	C	TE	MDR (R6AC)
AR58	AMP-S-SXT-NA-TE-C-CN-CIP-NOR	S,CN	NA,CIP,NOR	AMP		SXT	C	TE	MDR (R6AC)
AR59	AMP-SXT-NA-TE-C-CN-CIP	CN	NA,CIP	AMP		SXT	C	TE	MDR (R6AC)
AR60	AMP-S-SXT-NA-TE-C-CN-CIP-AMC	S,CN	NA,CIP	AMP,AMC		SXT	C	TE	MDR (R6AC)
AR61	AMP-S-SXT-NA-TE-C-CIP-AMC-CRO-NOR	S	NA,CIP,NOR	AMP,AMC	CRO	SXT	C	TE	MDR (R7AC)

AMP: Ampicillin, S: Streptomycin, SXT: trimethoprim-sulfamethoxazole, NA: Nalidixic, AMC: Amoxicillin clavulanic acid, TE: Tetracycline, C: Chloramphenicol, CN: Gentamicin, CIP: Ciprofloxacin, CRO: Ceftriaxone, NOR: Norfloxacin, MDR (R3AC): multi-drug resistance (resistance to 3 antibiotic classes); MDR (R4AC): multi-drug resistance (resistance to 4 antibiotic classes); MDR (R5AC): multi-drug resistance (resistance to 5 antibiotic classes); MDR (R6AC): multi-drug resistance (resistance to 6 antibiotic classes); MDR (R7AC): multi-drug resistance (resistance to 7 antibiotic classes).

Table S2. Primer lists for virulence genes.

Pathotype*	Primer name	Primer sequence (5′-3′)	Product size (bp)	References
E. coli	*uidA*	F: GCGTCTGTTGACTGGCAGGTGGTGG	510	[46]
		R: GTTGCCCGCTTCGAAACCAATGCCT		
STEC	*stx1*	F: CAGTTAATGTGGTGGCGAAGG	348	[46]
		R: CACCAGACAATGTAACCGCTG		
	stx2	F: ATCCTATTCCCGGGAGTTTACG	584	[46]
		R: GCGTCATCGTATACACAGGAGC		
EPEC	*bfp*	F: GGAAGTCAAATTCATGGGGGTAT	300	[46]
		R: GGAATCAGACGCAGACTGGTAGT		
	eae	F: TCAATGCAGTTCCGTTATCAGTT	482	[46]
		R: GTAAAGTCCGTTACCCCAACCTG		
ETEC	*It*	F: GCACACGGAGCTCCTCAGTC	218	[46]
		R: TCCTTCATCCTTTCAATGGCTTT		
EIEC	*virF*	F: AGCTCAGGCAATGAAACTTTGAC	618	[46]
		R: TGGGCTTGATATTCCGATAAGTC		
EAEC	*aafII*	F: CACAGGCAACTGAAATAAGTCTGG	378	[46]
		R: ATTCCCATGATGTCAAGCACTTC		
E. coli O157:H7	*eaeAO157:H7*	F: AAGCGACTGAGGTCACT	473	[46]
		R: ACGCTGCTCACTAGATGT		
Hemolysin	*hlyA*	F: GTCTGCAAAGCAATCCGCTGCAAATAAA	561	[51]
		R: CTGTGTCCACGAGTTGGTTGATTAG		
	sheA	F: GAGGCGAATGATTATGACTG	920	[51]
		R: ACTTCAGGTACCTCAAAGAG		
	ehxA	F: CCGTATCTTATAATAAGACGG	670	[27]
		R: CCTCCTTCATCTGCAATTG		

*STEC: Shiga toxin–producing *E. coli*, EPEC: Enteropathogenic *E. coli*, ETEC: Enterotoxigenic *E. coli*, EIEC: Enteroinvasive *E. coli*, EAEC: Enteroaggregative *E. coli*, EHEC: Enterohemorrhagic *E. coli*

Table S3. Primer lists for antibiotic-resistant genes.

Class of antibiotics	Primer name	Primer sequence (5′-3′)	T_A (°C)	Product size (bp)	References
Aminoglycosides	*aac (3)-IV*	F: GTGTGCTGCTGGTCCACAGC<br>R: AGTTGACCCAGGGCTGTCGC	50	627	[53]
	strA 2	F: CCTGGTGATAACGGCAATTC<br>R: CCAATCGCAGATAGAAGGC	55	546	[54]
Beta-lactam	bla_{TEM}	F: GAGTATTCAACATTTTCGT<br>R: ACCAATGCTTAATCAGTGA	50	857	[52]
Sulfonamides	*sul1*	F: TTCGGCATTCTGAATCTCAC<br>R: ATGATCTAACCCTCGGTCTC	50	822	[52]
	sul2	F: GCAGGCGCGTAAGCTGA<br>R: GGCTCGTGTGTGCGGATG	62.5	657	[55]
	sul3	F: ATTGATTTGGGAGCCGCTTC<br>R: AAAAGAAGCCCATACCCGGA	59.5	412	[55]
Phenicol	*catI*	F: AGTTGCTCAATGTACCTATAACC<br>R: TTGTAATTCATTAAGCATTCTGCC	50	547	[52]
	catII	F: GATTGACCTGAATACCTGGAA<br>R: CCATCACATACTGCATGATG	50	567	[56]
	florR	F: CGCCGTCATTCCTCACCTTC<br>R: GATCACGGGCCACGCTGTGTC	50	215	[56]
Tetracycline	*tetA*	F: GGTTCACTCGAACGACGTCA<br>R: CTGTCCGACAAGTTGCATGA	55	577	[57]

T_A: Annealing temperature (°C)

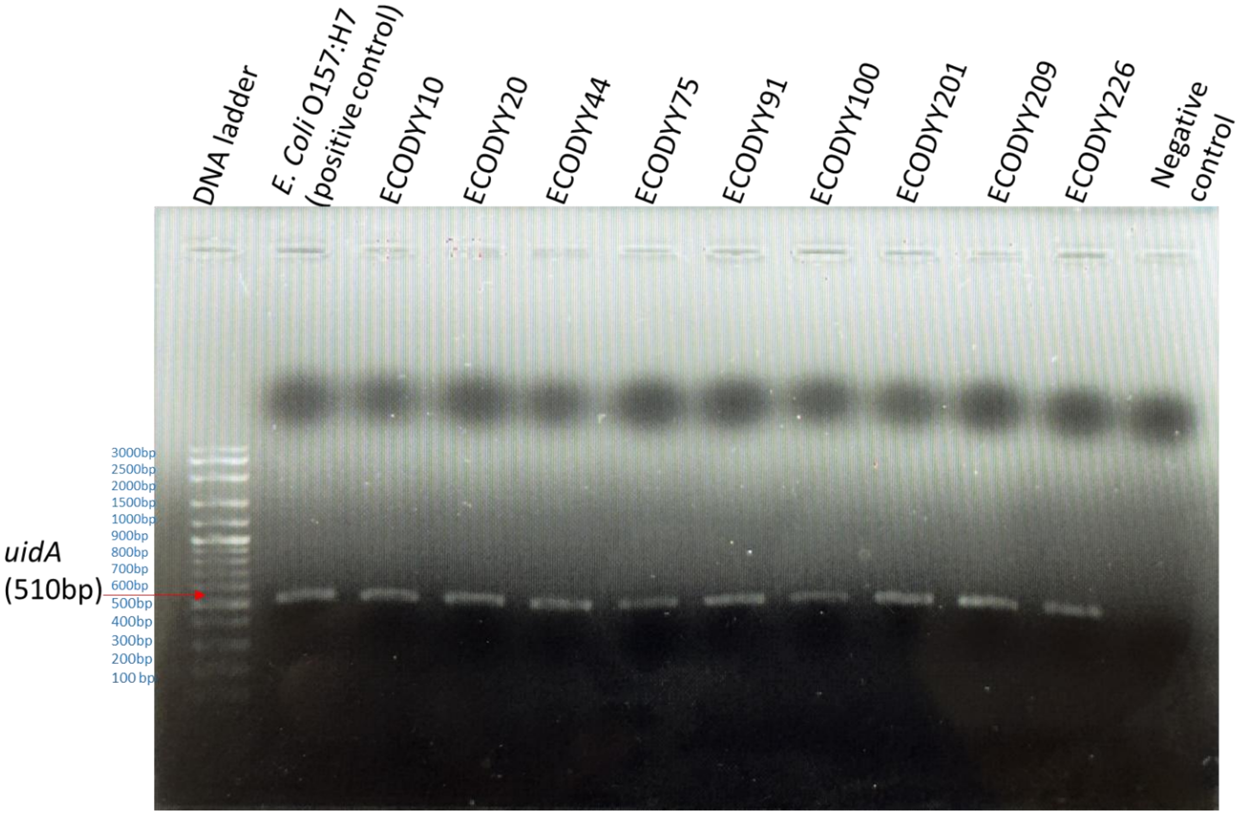

Figure S1. PCR confirmation of *E. coli* using the *uidA* gene
